# The Incidence of Acute Respiratory Infection Was Higher in the Older Adults with Lower Limb Fracture Who Receive Spinal Anesthesia Than Those Who Receive General Anesthesia

**DOI:** 10.3390/ijerph192114260

**Published:** 2022-11-01

**Authors:** Kuang-Ting Yeh, Wen-Tien Wu, Ru-Ping Lee, Jen-Hung Wang, Tsung-Ying Chen

**Affiliations:** 1 Department of Orthopedics, Hualien Tzu Chi Hospital, Buddhist Tzu Chi Medical Foundation, Hualien 970473, Taiwan; 2 Graduate Institute of Clinical Pharmacy, Tzu Chi University, Hualien 970374, Taiwan; 3 School of Medicine, Tzu Chi University, Hualien 970374, Taiwan; 4 Institute of Medical Sciences, Tzu Chi University, Hualien 970374, Taiwan; 5 Department of Medical Research, Hualien Tzu Chi Hospital, Buddhist Tzu Chi Medical Foundation, Hualien 970473, Taiwan; 6 Department of Anesthesiology, Hualien Tzu Chi Hospital, Buddhist Tzu Chi Medical Foundation, Hualien 970473, Taiwan

**Keywords:** general anesthesia, regional anesthesia, acute respiratory infection, surgery for lower limb fracture, nationwide retrospective cohort study

## Abstract

Introduction: Acute respiratory infection (ARI) can significantly reduce postoperative quality of life and impair the recovery of older adult patients with lower-limb fractures, and its relationship with methods of anesthesia remains inconclusive. Using data from the National Health Insurance Research Database (NHIRD) of Taiwan, this study examined the data of patients who received surgical management for lower-limb fractures and compared those who underwent general anesthesia (GA) with those who underwent regional anesthesia (RA) in terms of their incidence of acute upper and lower respiratory infection during the one-month postoperative period. The study also identified related risk factors. Material and Methods: Approximately two million patients were randomly sampled from the NHIRD registry. We identified and enrolled patients with lower-limb fractures who were over 60 years old and underwent GA or RA during surgeries conducted between 2010 and 2017. We divided these patients into two groups for further analysis. The outcome of this study was the development of ARI during the one-month postoperative period. Results: In total, 45,032 patients (GA group, 19,580 patients; RA group, 25,452 patients) with a mean age of 75.0 ± 8.9 years were included in our study. The incidence of postoperative ARI within one month of surgery was 8.0% (1562 patients) in the GA group and 9.5% (2412 patients) in the RA group, revealing a significant difference. The significant risk factors for the incidence of ARI were the application of RA for surgery, older age, hypertension, liver disease, and chronic obstructive pulmonary disease (COPD). A subgroup analysis revealed that the RA method was associated with a significantly higher ARI incidence relative to the GA method among patients aged between 60 and 80 years, among male patients, among the patients with or without any comorbidity and among the patients without COPD. Conclusion: The incidence of postoperative ARI within one month of surgery was higher among older patients with lower-limb fractures who received RA for surgery than among those who received GA for surgery. The other major risk factors for ARI were older age, hypertension, liver disease, and COPD. Therefore, we should focus on patients with a high risk of developing ARI, especially during the COVID-19 pandemic.

## 1. Introduction

Lower-limb fractures among the elderly population are increasing in incidence in this aging society and represent a rising number of complications with regard to disability in normal weight-bearing activities [1]. Postoperative respiratory complications were one of these complications and often occurred during the very early period of the fracture recovery stage [2,3]. Postoperative acute respiratory infection (ARI) may initiate a torrent of pulmonary complications and impair the recovery of older adults with lower-limb fractures. The anesthesia methods for surgery of the lower-limb fractures often directly influenced the respiratory function and may be related to the incidence of a postoperative pulmonary condition [4,5,6,7,8]. Anesthetic techniques can mainly be classified as general anesthesia (GA) and regional (caudal and neuraxial (spinal and epidural)) anesthesia (RA) [4]. Selecting a suitable anesthetic technique is a key decision that influences the surgical outcomes of lower-limb fractures, especially among older adults [5]. GA inhibits the central nervous system temporarily, which relaxes skeletal muscles and inhibits reflexes so that an operation can progress smoothly [6]. Tracheal intubation is a common airway management method that is employed during GA, and it may compromise the natural barrier of the respiratory tract and increase the risk of lung infection [7]. For the surgical management of geriatric hip fractures, GA may be associated with a higher risk of acute respiratory failure relative to spinal anesthesia [8]. The aging of the immune function of older adult patients may increase their susceptibility to bacterial or virus pulmonary infection due to various pathogens [9], especially among those who are older than 70 years, have a smoking habit, have diabetes, are receiving nasotracheal intubation, and are undergoing surgery with a duration of >180 min; all these factors are independent risk factors for lower respiratory tract infection [10]. Studies in the last five years have suggested that the incidence of pneumonia in patients who undergo GA for hip fracture surgery is similar to that in patients who undergo neuraxial anesthesia for this procedure [5,8,11]. Acute upper and lower respiratory infection may significantly reduce postoperative quality of life and impair the recovery of geriatric patients with lower-limb fractures [12]; however, the literature has mostly focused on the pulmonary complications after fracture surgery and few have mentioned the incidence of ARI during a short postoperative period, which may be related to the late respiratory complications and may need to be carefully prevented during the COVID-19 pandemic. This study aims to clarify how different anesthesia methods influence the incidence of ARI during the one-month postoperative period and to identify the related risk factors.

## 2. Materials and Methods

**Figure 1:**
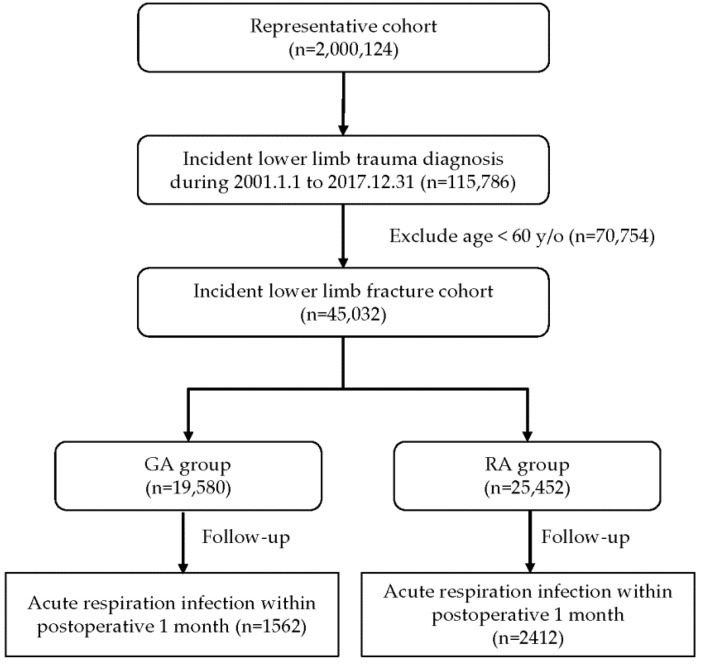
The flow chart of this nationwide retrospective cohort study.

This study was approved by the Research Ethics Committee of Hualien Tzu Chi Hospital, Buddhist Tzu Chi Medical Foundation (IRB 108-242-C). The requirement for written informed consent was waived by the Institutional Review Board of Hualien Tzu Chi Hospital. All the methods applied in this study were performed in accordance with the relevant guidelines and regulations. We used data from the Taiwan National Health Insurance Research Database (NHIRD), which was established by the National Health Insurance Administration, Ministry of Health and Welfare, and managed by the National Health Research Institutes; specifically, we obtained the data of patients who were older than 60 years and underwent GA for lower-limb trauma surgery between 1 January 2001, and 31 December 2017 (GA group). We also included the data of patients who were aged older than 60 years and underwent neuraxial anesthesia for the surgical treatment of lower-limb trauma (ICD-10, S70–S79, S80–S89, and S90–S99; ICD-9, 820–827, 835–838, 843–845, 890–897, 904.0, 904.1–5, 904.7–8, 916, 917, 924.0–5, 928, 929, 956, 957.9, 959.6, 959.7, V54.8, and V58.89) during the aforementioned time period (RA group); these patients were randomly sampled from a general population of 2,000,124 people with data in the NHIRD and assigned to the control group in Figure 1. The anesthesia type was identified using the payment codes as follows: 96020C–96022C for GA and 96005C–96008C for neuraxial anesthesia. We excluded the data of individuals who were younger than 60 years old and who had a prior diagnosis of ARI (ICD-10, J00–J06, J09–J18, and J20–J22; ICD-9, 034.0, 073.0, 115.95, 460–466, 480, 481, 482.0–4, 482.81–3, 482.89, 482.9, 483, 484.7–8, 485, 486, 487, 514, 517.1, and 519.8) during the one-month preoperative period before the index date on which they underwent anesthesia for surgery. The data of the two groups were collected and subjected to comparative analysis. The outcome of this study was ARI during the first month after operation. The disease codes of the comorbidities were based on the International Classification of Diseases, 9th Revision, Clinical Modification (ICD-9-CM) codes and ICD-10-CM codes, as listed in Table S1. All available data were used in this study, and no additional unpublished data were included.

All statistical analyses were performed using SAS version 9.4 and Stata version 16 (SAS Institute, Cary, NC, USA). Continuous variables were summarized as means and standard deviations, and categorical variables were listed as number of cases and percent values. Continuous between-group variables were compared using a Student’s *t*-test, and categorical variables were assessed using either a chi-squared test or a Fisher’s exact test. Logistic regression was adopted to evaluate the risk factors associated with acute respiratory problems. A *p* value of <0.05 and a standardized mean difference (SMD) of >0.1 were defined as statistically significant in this study.

## 3. Results

In total, the data of 45,032 patients (GA group, 19,580 patients; RA group, 25,452; male patients, 18,506 [41.1%]; female patients, 26,526 [58.9%]) were analyzed. The mean age of the patients examined in this study was 75.0 ± 8.9 years, and 14,971 (33.2%) of them were older than 80 years. There were 1409 (3.1%) patients having ARI within two weeks of surgery and 3974 (8.8%) patients having ARI within one month of surgery. The incidence of postoperative ARI within one month of surgery was 8.0 % (1562 patients) in the GA group and 9.5% (2412 patients) in the RA group, revealing a significant difference (*p* < 0.001; Table 1). The mean age of the RA group was older than that of the GA group. On the basis of the indicated *p* value and SMD, the two groups did not exhibit any significant difference in terms of gender and comorbidities (Table 1). The significant risk factors for the incidence of ARI within one month after surgery (Table 2) were the use of RA for surgery (adjusted hazard ratio [aHR], 1.17; 95% confidence interval [CI], 1.09–1.25; *p* < 0.001), older age (aHR, 1.16; 95% CI, 1.08–1.24; *p* < 0.001), hypertension (HTN; aHR, 1.16; 95% CI, 1.08–1.24; *p* < 0.001), liver disease (aHR, 1.20; 95% CI, 1.05–1.36; *p* = 0.006), and chronic obstructive pulmonary disease (COPD; aHR, 1.73; 95% CI, 1.57–1.90; *p* < 0.001). A subgroup analysis (results presented in Table 3) revealed that the GA method was associated with a significantly higher incidence of ARI relative to the RA method among the patients aged between 60 and 80 years (aHR, 1.22; 95% CI, 1.12–1.32; *p* < 0.001), male patients (aHR, 1.27; 95% CI, 1.14–1.41; *p* < 0.001), female patients (aHR, 1.10; 95% CI, 1.01–1.20; *p* = 0.036), patients with any comorbidity (aHR, 1.14; 95% CI, 1.01–1.29; *p* = 0.037), patients with any comorbidity (aHR, 1.18; 95% CI, 1.08–1.28; *p* < 0.001), and patients without COPD (aHR, 1.17; 95% CI, 1.09–1.25; *p* < 0.001). The effect of the anesthesia method on ARI incidence within one month of surgery was significantly greater in the male patients than in the female patients (*p* value for interaction, 0.020; Table 3). The significant risk factors for the incidence of ARI (Table S2) were male (aHR, 1.12; 95% confidence interval [CI], 1.00–1.25; *p* = 0.050), the use of RA for surgery (adjusted hazard ratio [aHR], 1.28; 95% confidence interval [CI], 1.14–1.42; *p* < 0.001), HTN (aHR, 1.22; 95% CI, 1.09–1.37; *p* < 0.001), and COPD (aHR, 1.55; 95% CI, 1.33–1.81; *p* < 0.001). The subgroup analysis (results presented in Table S3) revealed that the GA method was associated with a significantly higher incidence of ARI within two weeks after surgery relative to the RA method among the patients aged between 60 and 80 years (aHR, 1.25; 95% CI, 1.09–1.43; *p* = 0.001), the patients aged more than 80 years (aHR, 1.33; 95% CI, 1.09–1.61; *p* = 0.004), male patients (aHR, 1.27; 95% CI, 1.07–1.50; *p* = 0.005), female patients (aHR, 1.28; 95% CI, 1.10–1.48; *p* = 0.001), patients without any comorbidity (aHR, 1.29; 95% CI, 1.05–1.58; *p* = 0.015), patients with any comorbidity (aHR, 1.27; 95% CI, 1.11–1.45; *p* < 0.001), and patients without COPD (aHR, 1.29; 95% CI, 1.14–1.45; *p* < 0.001). The effect of the anesthesia method on ARI incidence within two weeks of surgery was not affected by the other risk factors based on the *p* value for the interaction method.

## 4. Discussion

Postoperative respiratory complications were common and had an estimated incidence of 3–7.9% among patients who underwent general surgery [13,14], including reintubation, acute respiratory failure, pulmonary edema, pneumonia, and atelectasis. Postoperative lower respiratory tract infections also appeared to be a common complication after GA with tracheal intubation; such infections accounted for more than 70% of the cases involving clinical nosocomial infections, especially among older adults [15,16,17]. For older adults who underwent surgery for hip fracture, the application of RA appeared to result in fewer cardiovascular and respiratory tract complications relative to the GA method [18,19]. Desai et al. suggested that RA techniques should be applied when possible because they can reduce the risk of mortality or complications within 90 days of surgery for older adults with hip fractures. In this study, we discovered that the incidence of ARI within two weeks or one month of surgery was higher in the RA group than in the GA group among the older patients who underwent surgery for lower-limb fractures. This correlation was more significant among the patients aged between 60–80, and patients without COPD. This raises a concern regarding the quality of care given to patients who underwent RA. Because RA is regarded to be a safer method relative to GA, surgeons may place less emphasis on protective measures (e.g., respiratory protection, use of warming blankets, and emotion regulation techniques) for patients undergoing RA than for those undergoing GA. Furthermore, patients who undergo RA must stay in bed for ≥6 h after surgery, whereas patients who undergo GA can wake up and commence normal activities if they do not exhibit contraindications. Cough reflex sensitivity may also possibly be influenced by spinal anesthesia and impair the postoperative respiratory function to cause further acute infection [20]. RA All of the aforementioned reasons can increase the risk of ARI during the one-month postoperative recovery period after anesthesia surgery. An increasing number of randomized controlled trial studies are reporting that the choice of anesthesia method for hip fracture surgery does not influence the occurrence of postoperative delirium, cognitive dysfunction, within-30-day mortality, and 60-day survival of ambulation in older patients [5,19,21]; these findings may be attributed to improvements in ventilatory methods and respiratory care during GA [22]. Thus, for older patients undergoing surgery for lower-limb fractures, the respiratory conditions and mobility of those who are undergoing RA should be closely monitored during and after surgery in addition to the implementation of measures for preventing GA-related complications. Although minor complications such as ARI generally do not directly cause the death of an older patient, they can still affect their recovery status and postoperative quality of life.

In our study, the other major risk factors for postoperative ARI within one month of surgery were older age, HTN, liver disease, and COPD. These findings are consistent with those of a 2019 study that investigated the independent risk factors for postoperative respiratory failure [23]. HTN can influence pulmonary circulation, whereas liver disease and COPD can negatively influence the immune response to environmental pathogens in a hospital or community setting; all of these factors can increase the vulnerability of older patients to respiratory infection, especially during the acute period around surgery. These comorbidity factors can also negatively influence the functional recovery of older patients with fractures and indirectly cause acute respiratory problems when the recovery of the lower extremities is slow [24].

The study has four limitations. First, we could not retrieve data pertaining to the ARI severity of the patients included because the NHIRD does not store symptom- and pathogen-related information. Second, we could neither clarify the details of the anesthesia processes such as temperature regulation and airway protection method nor locate detailed information on anesthesia medicine and the target level for regional anesthesia. Third, we could not retrieve data pertaining to the smoking habits of the patients included and the severity of environmental air pollution. Fourth, we cannot differentiate the patients with American Society of Anesthesiologists physical status classification because the NHIRD does not have this information, and we used information on the comorbidity of patients related to anesthesia risk as the risk factor instead for analysis. Despite the aforementioned limitations, the present study is the first to highlight the incidence of postoperative ARI within one month of surgery among older adults who underwent surgery for lower-limb fractures under different anesthesia methods. In a 2021 population-cohort study among more than eight million patients from United Kingdom, it was revealed that the patients with pre-existing respiratory disease had a modestly increased risk of severe COVID-19, especially those with asthma, interstitial lung disease or lung cancer [25]. ARI is generally a minor complication that is often neglected; however, it can cause severe respiratory problems, prolong normal rehabilitation, and increase the risk of exposure to severe pathogens such as SARS-CoV-2, the virus that causes COVID-19. For older patients who are undergoing RA for lower-limb fracture surgery to reduce their ARI risk, the primary protection measure should be the provision of excellent perioperative care.

## 5. Conclusions

The incidence of postoperative ARI within one month of surgery was higher in the older adult patients with lower-limb fractures who underwent RA for surgery than in those who underwent GA for surgery. The other major risk factors for ARI were older age, HTN, liver disease, and COPD. Relative to the other subgroups, the male patients, the patients aged between 60 and 80 years, and the patients without COPD had a higher incidence of postoperative ARI within one month of surgery when they underwent RA for surgery. During public health emergencies such as the COVID-19 pandemic, we should focus on patients with a high risk of developing ARI to prevent postoperative respiratory complications.

## Figures and Tables

**Table 1:** Demographics of the GA and SA groups of the patients receiving surgery for incidents of lower-limb fractures (*n* = 45,032).

Demographics	GA	RA	Total	*p* Value	SMD
N	19,580	25,452	45,032		
Age	73.8 ± 8.7	75.9 ± 8.9	75.0 ± 8.9	<0.001 *	0.24
Age group	-	-	-	<0.001 *	0.24
60–80 y/o	14,101(72.0%)	15,960(62.7%)	30,061(66.8%)		
≥80 y/o	5479(28.0%)	9492(37.3%)	14,971(33.2%)		
Gender	-	-	-	<0.001 *	0.08
Male	7845(40.1%)	10,661(41.9%)	18,506(41.1%)		
Female	11,735(59.9%)	14,791(58.1%)	26,526(58.9%)		
Comorbidities					
Hypertension (%)	9733(49.7%)	13,245(52.0%)	22,978(51.0%)	<0.001 *	0.05
Diabetes (%)	5073(25.9%)	6727(26.4%)	11,800(26.2%)	0.214	0.01
Dyslipidemia (%)	3616(18.5%)	4219(16.6%)	7835(17.4%)	<0.001 *	−0.05
Liver disease (%)	1287(6.6%)	1436(5.6%)	2723(6.1%)	<0.001 *	−0.04
Chronic renal faliure (%)	1102(5.6%)	1355(5.3%)	2457(5.5%)	0.161	−0.01
Chronic obstructive pulmonary disease (%)	1593(8.1%)	2740(10.8%)	4333(9.6%)	<0.001 *	0.09
Acute respiratory infection in postoperative 1 month (%)	1562(8.0%)	2412(9.5%)	3974(8.8%)	<0.001 *	0.05

Data are presented as *n* or mean ± standard deviation. * *p*-Value < 0.05 was considered statistically significant after test.

**Table 2:** Risk factors associated with the incidence of acute respiratory infection within postoperative 1 month (*n* = 45,032).

Risk Factors Associated with Acute Respiratory Infection	Crude		Adjusted	
	OR (95% CI)	*p* Value	OR (95% CI)	*p* Value
Age group (≥80 y/o vs. 60–80 y/o)	1.24(1.16, 1.33)	<0.001 *	1.16(1.08, 1.24)	<0.001 *
Gender (Male vs. Female)	1.04(0.97, 1.11)	0.253	0.99(0.93, 1.06)	0.848
Anesthesia (RA vs. GA)	1.21(1.13, 1.29)	<0.001 *	1.17(1.09, 1.25)	<0.001 *
Hypertension vs. None	1.18(1.11, 1.26)	<0.001 *	1.16(1.08, 1.24)	<0.001 *
Diabetes vs. None	0.97(0.90, 1.05)	0.465	0.96(0.89, 1.05)	0.380
Dyslipidemia vs. None	0.92(0.84, 1.01)	0.052	0.92(0.84, 1.01)	0.091
Liver disease vs. None	1.19(1.05, 1.36)	0.007 *	1.20(1.05, 1.36)	0.006 *
Chronic renal failure vs. None	0.98(0.85, 1.14)	0.836	0.95(0.82, 1.10)	0.472
Chronic obstructive pulmonary disease vs. None	1.82(1.66, 1.99)	<0.001 *	1.73(1.57, 1.90)	<0.001 *

Data are presented as odds ratio (95% CI). * *p*-Value < 0.05 was considered statistically significant after test.

**Table 3:** Subgroup comparative analysis of different age, gender, with or without any comorbidity, and with or without chronic obstructive pulmonary disease group associated with the incidence of acute respiratory infection within postoperative 1 month (*n* = 45,032).

Subgroup Comparative Analysis	Crude OR (95% CI) (RA vs. GA)	*p* Value	Adjusted OR (95% CI) (RA vs. GA)	*p* Value	*p* for Interaction
Main Model	1.21(1.13, 1.29)	<0.001 *	1.17(1.09, 1.25)	<0.001 *	
Age group					0.082
60–80 y/o	1.24(1.14, 1.34)	<0.001 *	1.22(1.12, 1.32)	<0.001 *	
≥80 y/o	1.10(0.98, 1.23)	0.102	1.08(0.96, 1.21)	0.195	
Gender					0.020 *
Male	1.35(1.21, 1.50)	<0.001 *	1.27(1.14, 1.41)	<0.001 *	
Female	1.12(1.03, 1.22)	0.012 *	1.10(1.01, 1.20)	0.036 *	
With any of comorbidity ^#^					0.767
No	1.16(1.03, 1.32)	0.015 *	1.14(1.01, 1.29)	0.037 *	
Yes	1.22(1.12, 1.32)	<0.001 *	1.18(1.08, 1.28)	<0.001 *	
Chronic obstructive pulmonary disease					0.879
No	1.19(1.10, 1.28)	<0.001 *	1.17(1.09, 1.25)	<0.001 *	
Yes	1.18(0.98, 1.41)	0.076	1.17(0.97, 1.40)	0.091	

Multiple logistic regression model with adjustment for all baseline characteristics shown in Table 1. Data are presented as odds ratio (95% CI). * *p*-value < 0.05 was considered statistically significant after test. ^#^ comorbidity includes hypertension, diabetes mellitus, dyslipidemia, liver disease, chronic renal failure, or chronic obstructive pulmonary disease.

## Data Availability

All the available data were used in this study, and no additional unpublished data were analyzed.

## Supplementary Materials

The following supporting information can be downloaded at: https://www.mdpi.com/article/10.3390/ijerph192114260/s1, Table S1: ICD9 and ICD10 diagnosis codes of the medical comorbidities. Table S2: Risk Factors associated with the incidence of acute respiratory infection within postoperative two weeks. Table S3: Subgroup comparative analysis of different age, gender, with or without any comorbidity, and with or without chronic obstructive pulmonary disease group associated with the incidence of acute respiratory infection within postoperative two weeks.
