# Supplementary material for: The Incidence of Acute Respiratory Infection Was Higher in the Older Adults with Lower Limb Fracture Who Receive Spinal Anesthesia Than Those Who Receive General Anesthesia
Source: Int J Environ Res Public Health. 2022 Nov 1;19(21):14260. doi: 10.3390/ijerph192114260 (PMC9654406; doi:10.3390/ijerph192114260)
Supplement: Supplementary file 1 [file ijerph-19-14260-s001.zip › ijerph-1933707-supplementary.pdf]

Table S1. ICD9 and ICD10 diagnosis codes of the medical comorbidities.

Diagnosis	ICD-9-CM	ICD-10-CM
Hypertension	401-405	I10, I11, I12, I13, I15
Diabetes Mellitus	250	E10, E11, E13
Dyslipidemia	272.X	E78
Chronic renal failure	585.X	N184, N185, N186, N189
Liver disease	571.X	K70, K73, K74, K754, K760, K769, K7581, K768
Chronic obstructive pulmonary disease	491,492,496	J449

Abbreviations: ICD-9-CM: International Classification of Diseases, Ninth Revision, Clinical Modification; ICD-10-CM: International Classification of Diseases, Tenth Revision, Clinical Modification

Table S2. Risk Factors associated with the incidence of acute respiratory infection within postoperative 2 weeks (n = 45,032).

Risk factors associated with acute respiratory infection	Crude		Adjusted	
	OR (95% CI)	*p* value	OR (95% CI)	*p* value
Age group (≥80 y/o vs. 60-80 y/o)	1.12(1.01, 1.25)	0.041*	1.04(0.93, 1.17)	0.486
Gender(Male vs. Female)	1.15(1.04, 1.28)	0.008*	1.12(1.00, 1.25)	0.050*
Anesthesia(RA vs. GA)	1.31(1.17, 1.46)	<0.001*	1.28(1.14, 1.42)	<0.001*
Hypertension vs. None	1.21(1.09, 1.35)	<0.001*	1.22(1.09, 1.37)	<0.001*
Diabetes vs. None	0.90(0.79, 1.01)	0.083	0.86(0.75, 1.01)	0.055
Dyslipidemia vs. None	1.00(0.87, 1.15)	0.952	1.05(0.90, 1.21)	0.553
Liver disease vs. None	1.02(0.82, 1.28)	0.838	1.03(0.82, 1.28)	0.803
Chronic renal failure vs. None	0.92(0.72, 1.17)	0.484	0.89(0.70, 1.14)	0.351
Chronic obstructive pulmonary disease vs. None	1.67(1.44, 1.94)	<0.001*	1.55(1.33, 1.81)	<0.001*

Data are presented as Hazard ratio (95% CI). *p-value < 0.05 was considered statistically significant after test.

Table S3. Subgroup comparative analysis of different age, gender, with or without any comorbidity, and with or without chronic obstructive pulmonary disease group associated with the incidence of acute respiratory infection within postoperative 2 weeks (n = 45,032).

Subgroup comparative analysis	Crude OR (95% CI) (SA vs. GA)	*p* Value	Adjusted OR (95% CI) (SA vs. GA)	*p* Value	*P* for interaction
Main Model	1.31(1.17, 1.46)	<0.001*	1.28(1.14, 1.42)	<0.001*	
Age group					0.653
60-80 y/o	1.27(1.11, 1.45)	0.001*	1.25(1.09, 1.43)	0.001*	
≥80 y/o	1.35(1.11, 1.64)	0.002*	1.33(1.09, 1.61)	0.004*	
Gender					0.984
Male	1.32(1.12, 1.56)	0.001*	1.27(1.07, 1.50)	0.005*	
Female	1.29(1.12, 1.49)	0.001*	1.28(1.10, 1.48)	0.001*	
With any of comorbidity[#]					0.807
No	1.31(1.07, 1.60)	0.009*	1.29(1.05, 1.58)	0.015*	
Yes	1.30(1.14, 1.48)	<0.001*	1.27(1.11, 1.45)	<0.001*	
Chronic obstructive pulmonary disease					0.661
No	1.30(1.15, 1.46)	<0.001*	1.29(1.14, 1.45)	<0.001*	
Yes	1.22(0.90, 1.64)	0.194	1.18(0.88, 1.59)	0.272	

Multiple logistic regression model with adjustment for all baseline characteristics shown in Table 1. Data are presented as odds ratio (95% CI). **p*-value < 0.05 was considered statistically significant after test. [#]cormobidity: hypertension, diabetes mellitus, dyslipidemia, liver disease, chronic renal failure, or chronic obstructive pulmonary disease.
